# Optimized Sample Handling Minimizes Peptide Adsorption to Plastics to Enable High Sensitivity Evosep Based Chemical Proteomics

**DOI:** 10.1002/pmic.70154

**Published:** 2026-06-22

**Authors:** Amrita Date, Anne Schuhmacher, Jianan Lu, Daria Berezina, Edward William Tate, Anna Barnard, Jack William Houghton

**Affiliations:** ^1^ Imperial College London Department of Chemistry Molecular Sciences Research Hub London UK

## Abstract

Chemical proteomics approaches often yield low peptide recovery because they aim to enrich low‐abundance proteins. Poor sample handling further reduces recovery through peptide adsorption to plastic surfaces and losses due to vacuum evaporation. We systematically mapped these losses across buffers, volumes, plastics and pH, then developed an Evotip‐compatible handling protocol that minimizes adsorption and removes the need for vacuum evaporation. Losses to plastic adsorption and vacuum evaporation were most apparent at low inputs (< 200 ng) and with larger volumes (> 20 µL) but were also dependent on acidification as well as the buffer and plastic used. The optimized workflow: direct acidification of peptides, frozen storage if needed, and direct loading onto Evotips, resulted in up to ∼90‐fold gains versus workflows incorporating vacuum concentration steps, a common practice in proteomics sample preparation, when peptide input was limited to 10 ng. This optimized sample handling method enables high‐throughput chemical proteomics by reducing manual handling steps and enables the characterization of low abundance proteins previously lost during sample preparation.

## 1 Introduction

Chemical proteomics has become a cornerstone of modern drug discovery, enabling proteome‐wide mapping of small‐molecule interactions and functional annotation of previously uncharacterized proteins and their post‐translational modifications directly in complex biological systems [1, 2]. Many chemical proteomics workflows rely on the enrichment of probe‐labelled targets prior to LC–MS/MS analysis. Labelling is achieved through covalent warheads [3], photo‐crosslinking [4], or metabolic incorporation [5], and is followed by the pull‐down of labelled targets by leveraging enrichment moieties such as clickable tags. These strategies typically enrich only a minute sub‐fraction of the proteome, so the captured material often falls well below the detection limits of routine peptide quantification assays. Laboratories therefore concentrate peptides before LC–MS/MS analysis, typically by vacuum concentration. Dried peptides are then re‐solubilized in water with trifluoroacetic acid or formic acid as an additive, with or without acetonitrile depending on whether the peptides will be loaded onto a conventional nanoLC system or an Evosep One platform, with the latter gaining in popularity for high‐throughput applications [6].

Significant peptide loss can arise through adsorption to plastic surfaces and incomplete recovery from vacuum concentration. These losses are most problematic when peptide recovery is already expected to be low, such as with highly selective probe pull‐downs. This previously described problem [7, 8, 9], has hindered chemical proteomics studies. Some studies compensate with milligram‐scale inputs [10] or overexpression systems [11], approaches that consume large quantities of often precious probe or depart from a native biological context, respectively. Other studies have failed to use proteomics to identify probe‐target interactions at all [12]. Optimized, low‐loss sample‐preparation workflows are therefore required both for the identification of low abundance targets and for scaling down chemical proteomics workflows enabling their application to increasingly popular high‐throughput experiments [13].

The extent of peptide loss by vacuum concentration has been previously studied and peptide sequence, charge, hydrophobicity, and size have been shown to be relevant factors [14, 15, 16, 17]. However, most published adsorption studies have relied on bovine serum albumin or a handful of representative synthetic peptides, providing only limited insight into proteome‐wide behavior [14, 16]. Whilst a range of strategies have been developed for other low‐input proteomics workflows to avoid this problem, namely single cell proteomics, these are often impractical for chemical proteomics laboratories. Many strategies rely on specialized equipment such as microfluid platforms [18, 19, 20], automated sample preparation platforms [21, 22, 23] or ultra‐sensitive mass spectrometers [24, 25]. Others have high consumable costs related to isobaric labelling [26, 27, 28] or are incompatible with click‐chemistry capture and on‐bead manipulations [29, 30]. There is therefore a need for broadly accessible sample preparation workflows that minimize peptide loss while remaining fully compatible with chemical proteomics enrichment chemistries and mainstream instrumentation. In this study, we investigate the role of pH, volume, buffer, storage and plastic in peptide loss from vacuum concentration in a whole proteome context and develop an optimized handling protocol for compatibility with an Evosep chromatography system and chemical proteomics.

## 2 Results and Discussion

**FIGURE 1:**
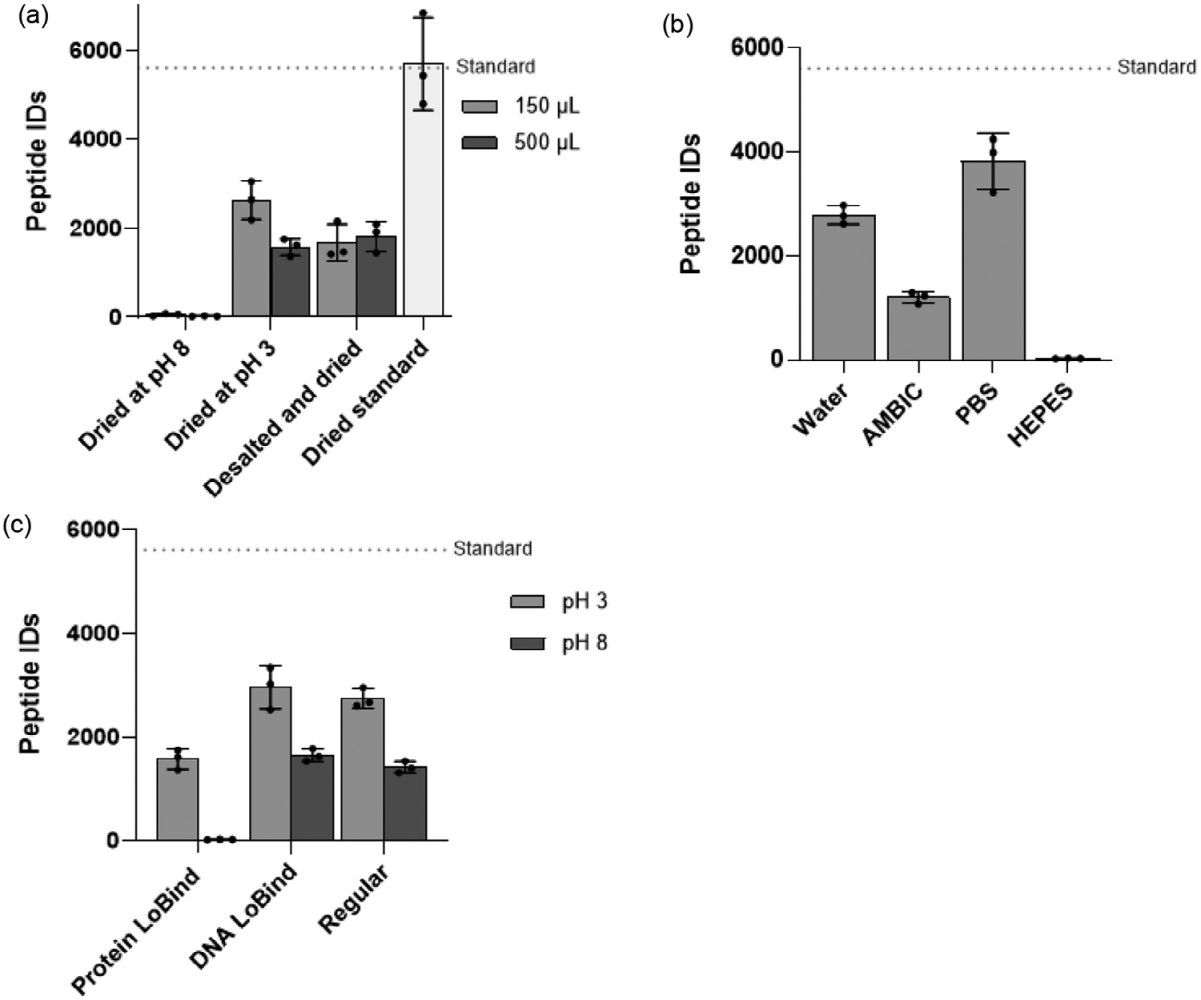
Number of peptides (mean ± standard deviation, *n* = 3) identified after drying 10 ng of HeLa standard under various conditions. Standard represents 10 ng HeLa standard frozen in 20 µL of 0.1% formic acid in water in a protein LoBind microcentrifuge tube. (a) Peptide in 150 or 500 µL of 50 mM HEPES pH 8.0 dried under different pH conditions or after desalting in protein LoBind plastic. The ‘dried standard’ was prepared by drying 10 ng HeLa standard in 20 µL of 0.1% formic acid in water. Dried peptides were redissolved in 20 µL 0.1% formic acid in water. (b) Peptide dried from 500 µL of various buffers at the standard working pH in Protein LoBind plastic. Dried samples were redissolved in 20 µL of formic acid in water at a concentration sufficient to give a final pH below 3. (c) Peptide dried from 500 µL of 50 mM HEPES pH 8.0 or acidified to pH <3.0 in various plastics. Dried samples were redissolved in 20 µL of formic acid in water at a concentration sufficient to give a final pH below 3.

We first examined how buffer volume and pH affected peptide recovery after sample drying using a SpeedVac concentrator. Commercially available pre‐digested HeLa lysate (10 ng) redissolved in 150 or 500 µL of 50 mM 2‐[4‐(2‐hydroxyethyl)piperazin‐1‐yl]ethanesulfonic acid (HEPES) buffer, pH 8.0 in an Eppendorf protein LoBind microcentrifuge tube was either (1) dried, (2) acidified to pH < 3 using formic acid and dried, or (3) desalted using a desalting spin column and dried. Samples were then redissolved in 0.1% formic acid at the time of loading onto Evotips for analysis using an Evosep One coupled with a timsTOF HT mass spectrometer. A representative chemical proteomics workflow involving drying samples at pH 8 (Figure 1a), resulted in a very low number of peptide identifications (average of 60 when dried from 150 µL and 25 when dried from 500 µL). The recovery increased significantly (p < 0.01) when the solutions were acidified prior to drying, resulting in an average of 2631 and 1579 peptide identifications when dried from 150 and 500 µL of buffer, respectively (Figure 1a). Lower peptide recovery (albeit not statistically significant) was observed when a higher volume of buffer was used; it was hypothesized that exposure to a larger surface area of plastic resulted in increased adsorption of the peptides, resulting in reduced recovery. This is consistent with previous reports of an increase in the adsorption of cationic peptides to polypropylene tubes with an increase in surface area: solution volume ratio and a decrease in peptide concentration [14]. Removal of salts from either 150 µL or 500 µL of buffer prior to drying resulted in a comparable number of identifications, which was unsurprising since peptides were eluted from the desalting spin columns in 300 µL of acidified solvent, resulting in a similar degree of exposure to the microcentrifuge tube plastic. Despite the improved recovery achieved through acidification, the peptide identifications were still less than 50% of the maximum, judged by the ‘standard’ (10 ng).

HeLa protein digest dissolved in 20 µL 0.1% formic acid, frozen at −20°C in a protein LoBind microcentrifuge tube. Drying and redissolving this 20 µL ‘standard’ solution did not result in significant peptide loss, likely due to the low volume. Protein IDs did reflect the same trends (Supporting Information).

Various strategies were explored to recover the peptides that were thought to be adsorbed on the surface of the plastic. It was observed that redissolution in 0.1% formic acid, the recommended solvent used for Evotip loading, was unable to buffer the solution to an acidic pH, which is required for optimal peptide binding to the Evotips. Therefore, dried peptides were redissolved in a higher concentration of 5% formic acid, however no improvement in the number of peptide identifications was seen. Attempts were also made to aid the redissolution of peptides through sonication and by altering the pH of the resolubilization buffer by redissolving the peptides in water and acidifying to pH < 3 after resolubilization. Neither of these approaches improved peptide recovery (Figure S3).

Next, the impact of buffer choice on peptide recovery was investigated. All buffers were tested at concentrations and pHs (7.5–8.0) used in proteomics protocols, and samples were dried in protein LoBind microcentrifuge tubes. Samples were redissolved in a concentration of formic acid required to reduce pH to below 3. Drying peptides in HEPES buffer continued to result in poor recovery. Drying from ammonium bicarbonate (AMBIC) buffer, while better than HEPES, resulted in fewer peptide identifications than from water (*p* < 0.01). Intriguingly, drying from phosphate buffered saline (PBS) resulted in a significantly greater number of peptide identifications than other buffers (*p* < 0.01), indicating that buffer salts can significantly impact the interactions of the peptides with plastic (Figure 1b).

The role that the choice of plastic plays in peptide recovery was also explored. Peptides were dried from a solution in 50 mM HEPES at pH 8 and pH 3 in protein LoBind, DNA LoBind, and regular microcentrifuge tubes. From all three plastics, significantly higher recovery (*p* < 0.01) was observed when the solutions were acidified to pH < 3 before drying. Protein LoBind tubes resulted in the lowest number of peptide identifications. DNA LoBind and the regular microcentrifuge tubes gave comparable recovery, with the DNA LoBind tubes performing slightly better and achieving the highest number of identifications at both pH's (Figure 1c). All three tubes are reported to be composed of uncoated polypropylene, with various additives lending protein or DNA low retention properties by reducing the hydrophobicity of the plastic [31]. The differences in peptide recovery attained by using different plastics reaffirms that peptide absorption is likely to be responsible for the low number of identifications.

**FIGURE 2:**
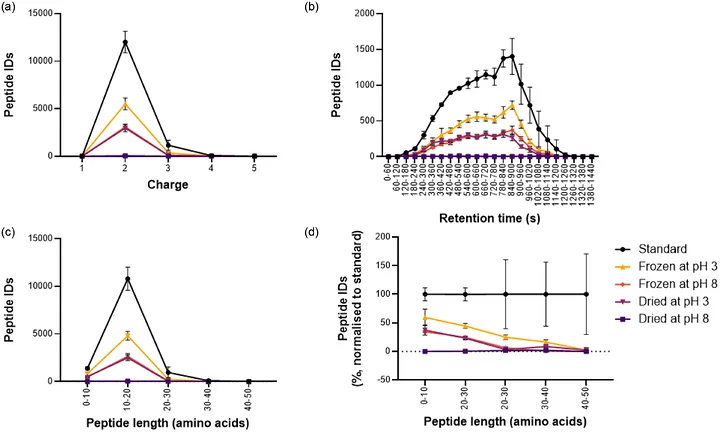
Properties of peptides identified after drying/freezing 10 ng HeLa standard in 500 µL 50 mM HEPES pH 8.0 or acidified to <3.0 in protein LoBind plastic. Standard represents 10 ng HeLa standard frozen in 20 µL of 0.1% formic acid in water in a protein LoBind microcentrifuge tube. (a) Number of peptides with different charges identified (mean ± standard deviation, *n* = 3). (b) Number of peptides identified (mean ± standard deviation, *n* = 3) across retention time ranges. (c) Number of peptides of different lengths identified (mean ± standard deviation, *n* = 3). (d) Number of peptides of each length identified, normalized to the corresponding number of identifications in the standard (mean ± standard deviation, *n* = 3).

To probe the nature of these interactions, the number of identifications for various peptide lengths, charge states and retention times were profiled. Peptide loss was observed across charge states, peptide lengths and retention times (Figure 2). Peptide length appeared to have the most defined impact on the number of peptide identifications, with the percentage loss being greatest for longer peptides (Figure 2d). No such patterns were observed with respect to peptide retention time or charge (Figure S4). Past studies too have revealed no significant patterns in peptide loss as a product of peptide charge, size, and regional charge distribution [16].

**FIGURE 3:**
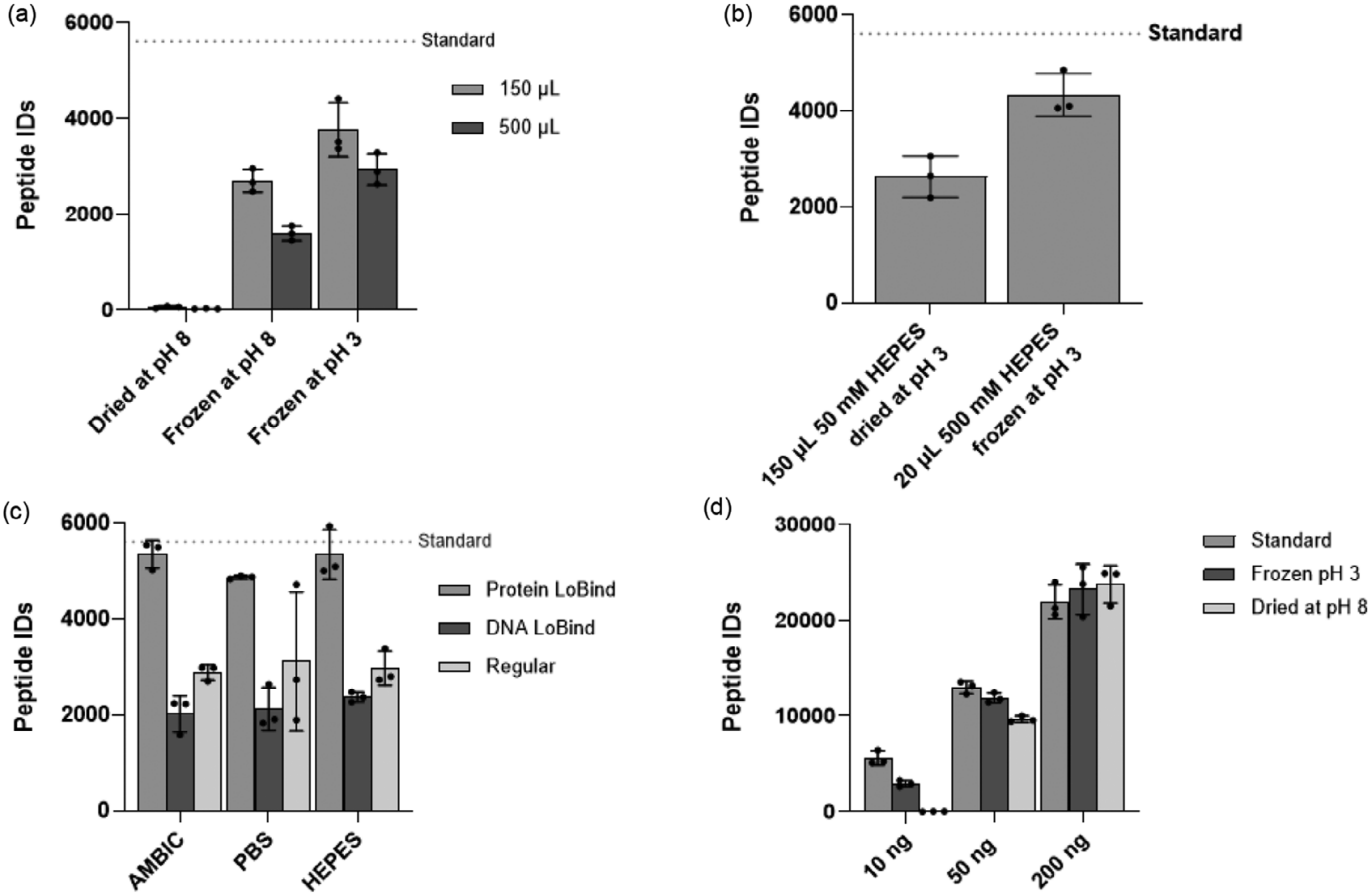
Number of peptides (mean ± standard deviation, *n* = 3) identified after freezing HeLa standard under various conditions. Standard represents 10 ng HeLa standard frozen in 20 µL of 0.1% formic acid in water in a protein LoBind microcentrifuge tube. (a) 10 ng peptide in 150 or 500 µL of 50 mM HEPES pH 8.0 dried or frozen under different pH conditions in protein LoBind plastic. Dried peptides were redissolved in 20 µL 0.1% formic acid in water. (b) 10 ng of HeLa standard dried or frozen in different concentrations of HEPES buffered to pH <3.0 in protein LoBind plastic. Dried peptides were redissolved in 20 µL 0.1% formic acid in water. Standard represents 10 ng HeLa standard frozen in 20 µL of 0.1% formic acid in water in a protein LoBind microcentrifuge tube. (c) 10 ng peptide frozen in 500 µL of commonly used buffers buffered to pH <3.0 using formic acid in various plastics. (d) Different peptide inputs frozen in 20 µL 0.1% formic acid, frozen in 500 µL of 50 mM HEPES buffered to pH<3 or dried from 500 µL of 50 mM HEPES pH 8.0 in protein LoBind plastic.

No drying condition achieved the maximum possible recovery seen with the ‘standard.’ We therefore tested removing the drying step and directly freezing (as this is usually carried out to store peptides between digestion and loading) the peptide solutions in 50 mM HEPES buffer at pH 8 and pH 3 in protein LoBind microcentrifuge tubes. They were thawed and loaded directly onto Evotips. Despite the manufacturer recommending that 20 µL of peptide solution should be loaded, Evotips have a capacity of 250 µL and this can be spun through within 1 min. For volumes greater than this, multiple cycles of samples loading, and centrifugation were required (it is important to note that the volume of wash steps after sample loading should be increased to the volume of the sample loaded to prevent the elution of any residual buffer salt with the peptides). This greatly improved peptide recovery, with freezing at pH < 3 producing the best results (Figure 3a). Higher buffer volume again caused a decrease in the number of peptides identified, suggesting that some binding with the plastics still occurred and highlighting the importance of keeping volumes low.

To understand whether this improvement in recovery was caused by the elimination of the drying step or if the concentration of buffer salts being loaded on the Evotips impacted their performance, peptide identifications after drying from 150 µL of 50 mM HEPES at pH 3, followed by redissolution in 20 µL of 0.1% formic acid versus directly freezing in 20 µL of 500 mM HEPES at pH 3 were compared. Direct freezing resulted in a greater number of identifications (*p* < 0.05), but less than that seen with the ‘standard’ (Figure 3b). Given that the solution was at a comparable volume and pH as the ‘standard,’ buffer concentration may also contribute to the observed effect. Some reports strongly caution against the freezing of peptide solutions, particularly for long‐term storage [17]. They demonstrate increased variability in results after storing peptide solutions in Eppendorf microcentrifuge tubes for 28 days, particularly in the case of hydrophobic peptides. Meanwhile, Hoofnagle et al. suggest that while short‐term storage as a high‐concentration solution can be acceptable, lyophilization is recommended for longer‐term storage (> 1 year) [32]. In this study, samples were stored as frozen peptide solutions for up to 1 week, and as such, a minimal variability was observed between replicates.

Following the improvemed recovery achieved through acidification and elimination of the drying step, buffers and plastics were screened to define an optimal sample handling protocol (Figure 3c). Conversely to what was observed while drying at pH 7.5–8, protein LoBind tubes performed the best, and DNA LoBind plastic resulted in the lowest number of peptide identifications when samples were frozen at an acidic pH. This was observed while using all three buffers, although differences among buffers were less pronounced. The effects observed were, once again, contrary to those observed while drying at a neutral to basic pH. Highest recovery was observed from HEPES and AMBIC, both closely approaching the maximum recovery set by the ‘standard’ with an average of 5355 and 5248 peptides identified, demonstrating a ∼90‐fold improvement over drying at pH 8 in 50 mM HEPES.

The differing performance of protein LoBind tubes under freezing versus drying conditions is likely a result of how the polypropylene surface is manufactured to reduce adsorption in aqueous environments through the formation of a water barrier to prevent protein–surface contact [33]. Under freezing conditions, although ice formation occurs, peptide adsorption to the tube surface is restricted due to maintenance of this barrier. In contrast, drying removes this aqueous environment entirely, increasing peptide concentration at interfaces and promoting structural perturbations that can enhance adsorption. Although the details of the differences between DNA and Protein LoBind are not explicitly disclosed by the manufacturer, we speculate that the Protein LoBind plastic is optimized to maximize the water barrier through a more hydrophilic surface which results in improved peptide recovery under freezing conditions compared to DNA LoBind or regular microtubes but promotes polar interactions between peptides and the surface in drying conditions resulting in reduced recovery.

The impact of protein input on peptide recovery was also investigated. The best and worst conditions identified were tested at three peptide inputs: 10, 50, and 200 ng (Figure 3d). Peptide losses were most differentiated at lower inputs (10 ng and 50 ng) and were insignificant at 200 ng. These results once again suggest peptide adsorption on the plastic surface. Once the surface is saturated, the rest of the peptides remain unbound and can be readily recovered, such that for a similar level of peptide adsorption, a smaller percentage loss is encountered at higher inputs. As a result, such losses have a significant impact on chemical proteomics experiments, particularly those conducted using highly selective probes.

Seeing as experiments up to this point had been conducted using pre‐digested peptides, the digest efficiency in each buffer was evaluated to ensure that the choice did not negatively impact other parts of a common cell lysate whole‐proteome digestion workflow [34]. The choice of buffer is known to impact the efficiency of tryptic digestion [35]. Tryptic digestion of HeLa lysate was carried out in HEPES, AMBIC or PBS buffers, and peptide solutions obtained were acidified and frozen in protein and DNA LoBind microcentrifuge tubes. Digestion in AMBIC buffer resulted in the highest peptide identifications, followed by PBS and HEPES (Figure S5a). However, peptides obtained from digestion in AMBIC also exhibited the highest percentage of missed cleavages (Figure S5b). The differences in the percentage of peptides identified with missed cleavages based on the type of plastic that the sample was stored in once again indicate that longer peptides containing a higher number of missed cleavages are preferentially adsorbed by the plastic.

Overall, these results demonstrate that elimination of vacuum concentration step at the end of commonly applied chemical proteomics workflows can enable a ∼90‐fold increase in the number of peptide identifications when peptide inputs are low (10 ng) [5]. This is particularly important for pulldown experiments where low levels of peptide (< 200 ng) are recovered. The use of Evotips for in‐line desalting allows loading of larger buffer volumes such that the need for sample concentration is eliminated. We find that the optimal conditions for sample handling post‐digestion involve acidifying samples to pH < 3 in protein LoBind microcentrifuge tubes, and freezing at −20°C if storage is required, prior to loading onto Evotips. Looking at digest efficiency and peptide recovery in conjunction, 25 mM AMBIC, pH 8 resulted in the highest number of peptide identifications. This optimized workflow can be applied to high‐throughput chemical proteomics as the greater sensitivity can enable the scaling down of experiments, potentially to 96‐well plate format, which reduces amounts of precious probe needed and allow the use of larger libraries of compounds.

## 3 Materials and Methods

### 3.1 Sample Preparation for Peptide Solutions

Lyophilized HeLa protein digest (Pierce) was redissolved in 0.1% formic acid in water to a concentration of 1 µg/µL. The desired amount of peptide for the experiment was diluted in buffer. Samples were prepared in triplicate.

### 3.2 Peptide Input

10, 50, or 200 ng HeLa protein digest was used for the experiments. Unless stated otherwise, the experiment used 10 ng of peptide.

### 3.3 Buffer

Peptide was diluted in 150 or 500 µL of the following buffers:

- 50 mM HEPES (Sigma‐Aldrich) pH 8.0
- 25 mM AMBIC (Fluka Analytical) pH 8.0
- 1X PBS (Sigma‐Aldrich) pH 7.5
- Water (Optima LC–MS grade, Fisher Chemical)
- 0.1% formic acid in water (Optima LC–MS grade, Fisher Chemical)

Unless stated otherwise, peptide was diluted in 500 µL of 50 mM HEPES pH 8.0.

### 3.4 Plastic

Samples were dried/frozen in one of the following 1.5 mL microcentrifuge tubes:

- Regular (Starlab TubeOne Cat no. S1615‐5550)
- Protein LoBind (Eppendorf tubes, Cat no. 0030108442)
- DNA LoBind (Eppendorf tubes, Cat no. 022431021)

Unless stated otherwise, protein LoBind microcentrifuge tubes were used.

### 3.5 Drying and Rehydration

Samples were dried down using a SpeedVac concentrator (Savant SDP1010, ThermoScientific) and stored at −20°C for up to one week. To rehydrate for loading onto Evotips (Evosep), 20 µL of solvent was added and samples were agitated at room temperature. One of the following solvents was used:

- 0.1% formic acid in water
- 5% formic acid (Optima LC–MS grade, Fisher Chemical) in water
- Water (in this case, samples were acidified to below pH 3 using formic acid before loading onto Evotips)

where specified, the sample was sonicated in a water bath at room temperature for 5 min to aid solubilization. Samples that were not dried were frozen at −20°C, thawed at room temperature and loaded directly onto Evotips.

### 3.6 Sample Preparation for Comparison of Digestion Conditions

HeLa cells, obtained from American Type Culture Collection, were cultured in low‐glucose Dulbecco's Modified Eagle Medium (Gibco), at 37°C in a 5% CO_2_ humidified incubator. Cells were grown to confluence and lysed using hot lysis buffer (1% sodium dodecyl sulphate in 50 mM HEPES pH 8.0 with 1x complete protease inhibitor cocktail) and sonicated on ice (5 s on, 5 s off for a total of 2 min). The protein content of the lysates was determined using a DC protein assay (Bio‐rad), and lysates were normalised to 2 mg/mL. To a 30 µg aliquot in a protein LoBind microcentrifuge tube, 10 mM tris(2‐carboxyethyl)phosphine (Bond‐Breaker TCEP solution, ThermoScientific 77720) and 40 mM chloroacetamide (SigmaAldrich) were added and samples were agitated for 10 min at room temperature. The sample was split into three different aliquots of 10 µg. Protein was precipitated by addition of 40 µL acetonitrile (HPLC grade, VWR). The samples were centrifuged to obtain a protein pellet, and the supernatant was discarded. The pellet was washed three times with 80% ethanol (HPLC grade, VWR). The supernatant was removed and a solution of trypsin (6.25 ng/µL, Sequencing grade modified trypsin, Promega V5111) in 25 µL of either 50 mM HEPES pH 8.0, 25 mM AMBIC pH 8.0, or 1x PBS pH 7.5 was added to the protein pellet. The samples were agitated overnight at 37°C. The samples were visually checked to ensure that the protein pellet had disappeared, and the peptide content of the digested samples was determined using a NanoDrop OneC (ThermoScientific). The samples were diluted in the respective buffer to a concentration of 10 ng/µL and acidified using formic acid to pH <3. The samples were divided into protein and DNA LoBind microcentrifuge tubes and frozen at −20°C. At the time of running, samples were thawed at room temperature and 20 µL was loaded onto Evotips. All samples were prepared in triplicate.

### 3.7 Evotip Loading

Evotips were equilibrated according to the manufacturer's instructions. Briefly, tips were washed with 20 µL of acetonitrile containing 0.1% formic acid, then centrifuged for 1 min at 800 g. Next, they were soaked in propanol for 1 min, and while still soaking, 20 µL of 0.1% formic acid was applied on top for a second wash, followed by centrifugation at 800 g for 1 min. Samples were then loaded up to 250 µL. If the sample volume exceeded 250 µL, the volume was split into two and loaded in two steps. After sample loading, the Evotips were washed twice with 0.1% formic acid with the volume used to load each sample, each time followed by centrifugation for 1 min at 800 g. Finally, 300 µL of 0.1% formic acid was added to each Evotip and spun down for 10 s to minimize solvent loss by evaporation.

### 3.8 LC–MS/MS

Peptides were analyzed by nanoLC–MS/MS using an Evosep One (Evosep) coupled with a timsTOF HT (Bruker) equipped with an 8 cm x 150 µm, 1.5 µm analytical column (Evosep). Peptides were separated by the Evosep 60SPD workflow (Analytical solvents A: 0.1% formic acid and B: acetonitrile plus 0.1% formic acid). Column was held at 40°C. Data were acquired in data‐dependent acquisition (DDA) PASEF mode with the following settings: m/z range from 100 m/z to 1700 m/z, ion mobility ranges from 1/K0 = 1.30–0.85 Vs/cm^2^ using equal ion accumulation and ramp times in the dual TIMS analyser of 100 ms each. The collision energy was lowered as a function of increasing ion mobility from 59 eV at 1/K0 = 1.6 Vs/cm^2^ to 20 eV at 1/K0 = 0.6 Vs/cm^2^. Isolation width was lowered as a function of decreasing m/s from 3 m/z at 800 m/z to 2 m/z at 700 m/z. Active exclusion was applied for 0.4 min. Each cycle consistent of 4 PASEF ramps (total cycle time 0.53 sec) with 2.75 ms measuring time allowed for each selected precursor. A 50 ng HeLa digest QC sample was run between batches of replicates to verify instrument performance.

### 3.9 Data Processing

ddaPASEF Bruker d files were processed using Fragpipe 22.0 with the built‐in “LFQ MBR” workflow with some modifications. Default settings, other than MaxLFQ min ions was set as 1 and match between runs was disabled, were used. Briefly, Bruker d files were searched using MSFragger [36] (version 4.0) with the following parameters: strict trypsin digestion; up to 2 missed cleavages allowed; precursor ion tolerance: 20 ppm; trimming of protein N‐terminal methionine; oxidation (M) and N terminal acetylation as variable modifications; carbamidomethylation (C) as a fixed modification; Human database (Downloaded from UniProt 13 July, 2023 containing 20588 proteins) with decoys (50%) and common contaminants added. MSFragger search results were processed using Percolator [37] (Version 3.6.4) for peptide‐spectrum match validation, followed by Philosopher [38] (Version 5.1.0) for protein and FDR filtering. Label‐free quantification values were calculated using the MaxLFQ algorithm using IonQuant [39] (Version 1.10.12) with match between runs disabled and min ions set as 1.

### 3.10 Data Analysis

The number of protein and peptide identifications for each sample were counted based on those with recorded intensities greater than 0. Results are reported as the mean ± standard deviation (*n* = 3). Graphs were plotted in Prism 10 (Graphpad). P values were determined from AOVA and Tukey's multiple comparison testing, performed in Prism 10 (Graphpad). Details of all comparisons made can be found in Tables S1–S7.

## Conflicts of Interest

E.W.T. is a founder and shareholder in Myricx Pharma and Siftr Bio. The other authors have no conflict of interest to declare.

## Supporting information




**Supporting File 1**: pmic70154‐sup‐0001‐TableS1.xlsx.

**Supporting File 2**: pmic70154‐sup‐0002‐FigureS1.docx.

## Data Availability

The mass spectrometry proteomics data have been deposited to the ProteomeXchange Consortium via the PRIDE partner repository with the dataset identifier PXD069322.
