# Supplementary material for: Optimized Sample Handling Minimizes Peptide Adsorption to Plastics to Enable High Sensitivity Evosep Based Chemical Proteomics
Source: Proteomics. 2026 Jun 22;26(10):16–23. doi: 10.1002/pmic.70154 (PMC13615481; doi:10.1002/pmic.70154)
Supplement: Supplementary file 1 [file PMIC-26--s001.docx]

**Supplementary Figures**

**Figure S1:** Number of proteins (mean ± standard deviation, n = 3) identified after drying 10 ng of HeLa standard under various conditions. Standard represents 10 ng HeLa standard frozen in 20 μL of 0.1% formic acid in water in a protein LoBind microcentrifuge tube. **(a)** Peptide in 150 or 500 μL of 50 mM HEPES pH 8.0 dried under different pH conditions or after desalting in protein LoBind plastic. The ‘dried standard’ was prepared by drying 10 ng HeLa standard in 20 μL of 0.1% formic acid in water. Dried peptides were redissolved in 20 μL 0.1% formic acid in water. **(b)** Peptide dried from 500 μL of various buffers at the standard working pH in Protein LoBind plastic. Dried samples were redissolved in 20 μL of formic acid in water at a concentration sufficient to give a final pH below 3. **(c)** Peptide dried from 500 μL of 50 mM HEPES pH 8.0 or acidified to pH <3.0 in various plastics. Dried samples were redissolved in 20 μL of formic acid in water at a concentration sufficient to give a final pH below 3.

**Figure S2:** Number of proteins (mean ± standard deviation, n = 3) identified after freezing HeLa standard under various conditions. Standard represents 10 ng HeLa standard frozen in 20 μL of 0.1% formic acid in water in a protein LoBind microcentrifuge tube. (a) 10 ng peptide in 150 or 500 μL of 50 mM HEPES pH 8.0 dried or frozen under different pH conditions in protein LoBind plastic. Dried peptides were redissolved in 20 μL 0.1% formic acid in water. (b) 10 ng of HeLa standard dried or frozen in different concentrations of HEPES buffered to pH <3.0 in protein LoBind plastic. Dried peptides were redissolved in 20 μL 0.1% formic acid in water. Standard represents 10 ng HeLa standard frozen in 20 μL of 0.1% formic acid in water in a protein LoBind microcentrifuge tube.  (c) 10 ng peptide frozen in 500 μL of commonly used buffers buffered to pH <3.0 using formic acid in various plastics. (d) Different peptide inputs frozen in 20 μL 0.1% formic acid, frozen in 500 μL of 50 mM HEPES buffered to pH<3 or dried from 500 μL of 50 mM HEPES pH 8.0 in protein LoBind plastic.

**Figure S3** Number of peptides (a) and proteins (b) (mean ± standard deviation, n = 3) identified after drying 10 ng of HeLa lysate standard from 500 μL of 50 mM HEPES pH 8.0 in a protein LoBind microcentrifuge tube and rehydrating in 20 μL of different concentrations of formic acid in water, with or without sonication to aid redissolution.

**Figure S4:** Properties of peptides identified after drying/freezing 10 ng HeLa standard in 50 mM HEPES pH 8.0 or acidified to <3.0 in protein LoBind plastic. Standard represents 10 ng HeLa standard frozen in 20 μL of 0.1% formic acid in water in a protein LoBind microcentrifuge tube. **(a)** Number of peptides identified across the retention time range, normalised to the corresponding number of identifications in the standard (mean ± standard deviation, n = 3). **(b)** Number of peptides of each charge state identified, normalised to the corresponding number of identifications in the standard (mean ± standard deviation, n = 3).

**Figure S5** Number of **(a)** peptides, **(b)** proteins and **(c)** missed cleavages (mean ± standard deviation) identified after preparing samples by digesting HeLa lysate in different buffers. The peptide mixtures obtained after digest were diluted to 0.01 μg/μL, buffered to pH <3.0 using formic acid, and frozen in either protein or DNA LoBind plastic.
